# Heterogeneity in the onwards transmission risk between local and imported cases affects practical estimates of the time-dependent reproduction number

**DOI:** 10.1098/rsta.2021.0308

**Published:** 2022-10-03

**Authors:** R. Creswell, D. Augustin, I. Bouros, H. J. Farm, S. Miao, A. Ahern, M. Robinson, A. Lemenuel-Diot, D. J. Gavaghan, B. C. Lambert, R. N. Thompson

**Affiliations:** ^1^ Department of Computer Science, University of Oxford, Oxford OX1 3QD, UK; ^2^ Mathematical Institute, University of Oxford, Oxford OX2 6GG, UK; ^3^ Roche Pharmaceutical Research and Early Development, Pharmaceutical Sciences, Roche Innovation Center Basel, Basel CH-4070, Switzerland; ^4^ Mathematics Institute, University of Warwick, Coventry CV4 7AL, UK; ^5^ Zeeman Institute for Systems Biology and Infectious Disease Epidemiology Research, University of Warwick, Coventry CV4 7AL, UK

**Keywords:** mathematical modelling, reproduction number, imported cases, branching processes, COVID-19, SARS-CoV-2

## Abstract

During infectious disease outbreaks, inference of summary statistics characterizing transmission is essential for planning interventions. An important metric is the time-dependent reproduction number (*R_t_*), which represents the expected number of secondary cases generated by each infected individual over the course of their infectious period. The value of *R_t_* varies during an outbreak due to factors such as varying population immunity and changes to interventions, including those that affect individuals' contact networks. While it is possible to estimate a single population-wide *R_t_*, this may belie differences in transmission between subgroups within the population. Here, we explore the effects of this heterogeneity on *R_t_* estimates. Specifically, we consider two groups of infected hosts: those infected outside the local population (imported cases), and those infected locally (local cases). We use a Bayesian approach to estimate *R_t_*, made available for others to use via an online tool, that accounts for differences in the onwards transmission risk from individuals in these groups. Using COVID-19 data from different regions worldwide, we show that different assumptions about the relative transmission risk between imported and local cases affect *R_t_* estimates significantly, with implications for interventions. This highlights the need to collect data during outbreaks describing heterogeneities in transmission between different infected hosts, and to account for these heterogeneities in methods used to estimate *R_t_*.

This article is part of the theme issue 'Technical challenges of modelling real-life epidemics and examples of overcoming these'.

## 1. Introduction

Mathematical and computational models have been used during the COVID-19 pandemic to infer changes in transmissibility and to plan public health measures [1–7]. An important metric for assessing the effectiveness of current interventions during outbreaks is the time-dependent reproduction number (*R_t_*—sometimes referred to informally as the ‘*R* number’), which represents the expected number of infections generated by someone infected at time *t* over the course of their infectious period [8–17]. This quantity varies during an outbreak in response to factors affecting transmission such as changes in public health measures, varying population immunity and pathogen evolution. If *R_t_* remains below one, the number of cases each day will decrease; if instead *R_t_* is persistently above one, the outbreak will grow. In the UK, the government has published estimates of *R_t_* throughout the COVID-19 pandemic [18] alongside other values such as estimates of the epidemic growth rate and daily numbers of new reported cases, hospitalizations and deaths.

Different formal definitions of *R_t_* have been proposed, most notably the instantaneous reproduction number and the case reproduction number [19]. The instantaneous reproduction number represents the expected number of infections generated (over the course of their infectious period) by someone who is infected at time *t* if transmission conditions do not change in the future (i.e. this quantity is a measure of instantaneous transmissibility). The case reproduction number, on the other hand, reflects the expected number of infections generated by someone who is infected at time *t* but accounts for changes in transmissibility that occur after time *t* (e.g. the subsequent introduction of public health measures). The instantaneous reproduction number has been proposed as the most appropriate definition to use for real-time inference, as this quantity reflects current transmissibility and does not require future changes in transmission conditions to be known [11]. For that reason, we use this definition of *R_t_* for our analyses in this manuscript.

A range of methods has been developed for estimating *R_t_* from outbreak data [11,12,20,21]. Two common approaches are the Cori method [8,9] and the Wallinga–Teunis method [22], which involve inferring the value of *R_t_* from disease incidence time-series (i.e. time-series describing the number of new cases every day) and an estimate of the serial interval distribution (representing the time period between successive cases; specifically, the difference between the symptom onset times of infectors and infectees). Irrespective of the precise approach used to infer *R_t_*, estimates can be updated and tracked as additional data become available during an outbreak.

Recent developments in the theory of *R_t_* estimation include accounting for reporting delays [7] and considering the impacts of temporal changes in the serial interval [23]. Another consideration is the potential for heterogeneity in *R_t_* between different subgroups in the population. The COVID-19 pandemic has highlighted that individuals in different settings (e.g. care homes as opposed to the wider population [24]) or with different characteristics (e.g. different ages [10,25–28] or vaccination statuses [29,30]) face different risks of both becoming infected and transmitting the virus. Shortly before the COVID-19 pandemic, the Cori method was extended to account for differences in the source locations of local and imported cases [9], but with an assumption that the expected numbers of onwards transmissions from each local case and each imported case are identical. With that assumption, that work illustrated that failing to differentiate between local and imported cases can lead to overestimation of the number of local infections and therefore overestimation of *R_t_* [9].

Apart from their different origins, local and imported cases can differ in other ways. The risk of onwards transmission from an imported case may be different to the risk from a local case [31]. Imported cases may have visited regions with high case numbers and therefore respond more quickly to early signs of disease, isolating as soon as symptoms develop. This effect might be especially pronounced when a pathogen has first arrived in the local host population, when the infection risk may be higher outside the local population than within it. Imported cases may also be subject to increased testing for infection or pre-emptive home quarantine following travel, thereby lowering the risk of onwards transmission [32]. On the other hand, individuals who travel frequently may be likely to have more contacts with others than those who do not, potentially leading to a higher risk of onwards transmission for imported cases. For example, business travellers may participate in large numbers of meetings, thereby coming into contact with many other people. In either situation, an assumption that *R_t_* is identical for both local cases and imported ones, as made previously [9], is not always appropriate.

**Figure 1.**
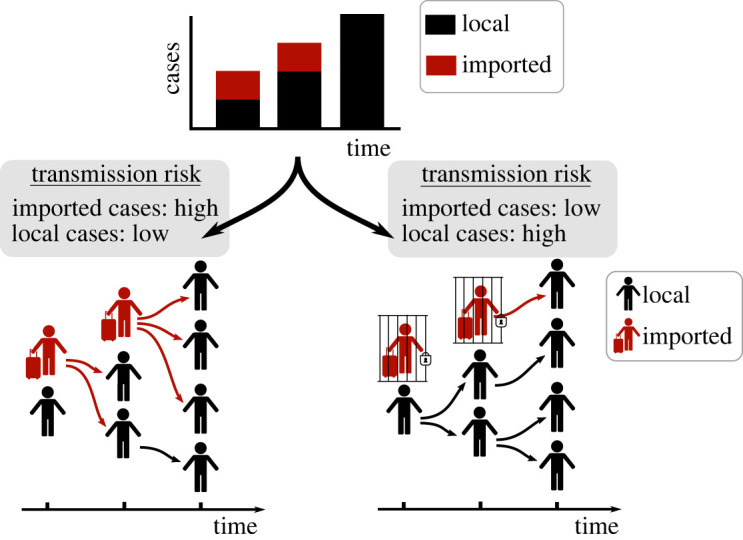
A disease incidence time-series dataset can be generated by different combinations of transmission risks from imported and local cases. In the first scenario (bottom left), observed cases are mostly due to infections by imported cases, whereas in the second scenario (bottom right), observed cases are mostly due to infections by local cases. In the bottom panels, red arrows represent infections generated by imported cases and black arrows represent infections generated by local cases. An individual who is infected by an imported case is classified as a local case, since they have themselves been infected locally. Despite the same overall incidence, the two scenarios shown correspond to different risks of sustained local transmission (the risk of sustained local transmission is higher in the second scenario—bottom right), with implications for public health measures. (Online version in colour.)

In principle, the same disease incidence time-series can occur with different divisions of the transmission risk between local and imported infectors (figure 1). This has implications for pathogen control, since a scenario with substantial local transmission requires localized public health measures to disrupt chains of transmission and prevent spread. By contrast, a scenario with high transmission from imported cases may motivate travel restrictions to prevent importations. Here, we modify the Cori method to allow local and imported cases to have unequal risks of generating new infections. We analyse disease incidence time-series recorded during the COVID-19 pandemic in different locations. Our main goal is not to provide a novel methodological approach for estimating *R_t_*, but rather to explore as simply as possible the potential consequences for estimates of *R_t_* of failing to account for differences in the onwards transmission risk from local and imported cases. To allow other researchers to repeat our analyses for similar data, we provide an open-source Python software library including a user-friendly web interface (https://sabs-r3-epidemiology.github.io/branchpro). Our research demonstrates the importance of accounting for differences in the transmission risk between imported and local cases. More widely, it indicates that careful consideration of heterogeneity in the transmission risk between population subgroups may be necessary to make robust public health policy decisions.

## 2. Methods

### (a) Inference of the time-dependent reproduction number

We modify the Cori method for estimating *R_t_* [8,9] to account for differences in the onwards transmission risk between cases that arise locally compared with those originating elsewhere. In the underlying transmission model, new cases occur according to a time-varying branching process in which each local case is assumed to generate *R_t_* new infections on average, and each imported case is expected to generate ε*R_t_* new infections on average, where ε ≥ 0 indicates the relative transmission risk from an imported case compared with a local case. Here, we assume that *R_t_* is the instantaneous reproduction number [8,9], representing the expected number of cases that an individual infected at time *t* is likely to generate over the course of their infection assuming that future pathogen transmissibility is fixed at the current level. Our focus is on estimating the extent of local transmission (i.e. the local time-dependent reproduction number [21]) characterized by *R_t_*. As has been proposed previously [21], the value of *R_t_* therefore reflects the potential for local transmission of the pathogen (rather than being an averaged quantity across both local and imported cases). Here, ε < 1 means that an imported case is responsible for fewer infections (on average) than a local case, whereas ε > 1 indicates that an imported case generates more infections.

The total number of new cases arising at time step *t* can be split according to the sources of infection, $I_{t}=I_{t}^{\text{loc}}+I_{t}^{\text{imp}}$, where $I_{t}^{\text{loc}}$ represents the number of new cases who were infected within the local population and $I_{t}^{\text{imp}}$ represents the number of new cases who were infected elsewhere. The expected number of local cases at time step *t* (measured in days) is then given by

$$\text{E}(I_{t}^{\text{loc}}|{\left\{I_{k}^{\text{loc}}\right\}}_{k=0}^{t-1},{\left\{I_{k}^{\text{imp}}\right\}}_{k=0}^{t-1},\varepsilon ,R_{t},\text{w})=R_{t}\overset{t}{\underset{s=1}{\sum }}(I_{t-s}^{\text{loc}}+\varepsilon I_{t-s}^{\text{imp}})w_{s}.$$

In this expression, the vector **w** is the (discrete) serial interval distribution with entries *w*_s_ (which characterizes the times between successive cases in a chain of transmission; *w*_1_ is the probability that the serial interval is one day, *w*_2_ is the probability that the serial interval is two days, and so on).

We define the transmission potential at time step *t* to represent the expected number of local cases arising at time step *t* if *R_t_* = 1. Thus, the transmission potential at time step *t* is given by $\Lambda_{t}(\text{w},\varepsilon )=\sum_{s=1}^{t}(I_{t-s}^{\text{loc}}+\varepsilon I_{t-s}^{\text{imp}})w_{s}$. We assume that the number of local cases in time step *t* is drawn from a Poisson distribution with mean *R_t_*Λ*_t_*(**w**, ε). Hence, the probability of observing the local incidence ${\left\{I_{k}^{\text{loc}}\right\}}_{k=t-\tau }^{t}$ over a time window including *τ* + 1 days (assuming that *R_t_* is constant during that time window), conditional each day on all previous incidence data, is given by

$$\text{P}({\left\{I_{k}^{\text{loc}}\right\}}_{k=t-\tau }^{t}\text{|}{\left\{I_{k}^{\text{loc}}\right\}}_{k=0}^{t-\tau -1},{\left\{I_{k}^{\text{imp}}\right\}}_{k=0}^{t-1},\varepsilon ,R_{t},\text{w}\text{) }=\overset{t}{\underset{k=t-\tau }{\prod }}\frac{{\left(R_{t}\Lambda_{k}(\text{w},\varepsilon )\right)}^{I_{k}^{\text{loc}}}\text{exp}(-R_{t}\Lambda_{k}(\text{w},\varepsilon ))}{I_{k}^{\text{loc}}!}.$$

Data describing daily numbers of imported cases enter this expression through Λ*_t_*(**w**, ε). The model therefore reflects how local cases arise using information about historical numbers of local and imported cases.

Assuming a gamma distributed prior for *R_t_*, the posterior distribution for *R_t_* over the time window [*t* − *τ*, *t*], conditional on **w**, ε and the observed incidence data (denoted *p*(*R_t_*|**w**, ε, ***I***_≤_***_t_***)—we represent this by *p*(·) rather than **P**(·) since the posterior is a continuous probability density function), is also a gamma distribution due to prior-likelihood conjugacy (see Cori *et al.* [8] and Thompson *et al.* [9] for further details). Specifically,

$$p(R_{t}|\text{w},\;\varepsilon ,I_{\leq t})=\text{gamma}\left(R_{t},\alpha +\overset{\tau }{\underset{k=0}{\sum }}I_{t-k}^{\text{loc}},\beta +\overset{\tau }{\underset{k=0}{\sum }}\Lambda_{t-k}(\text{w},\varepsilon )\right),$$

where for notational convenience, here and above we have combined the disease incidence data into the variable $I_{\leq t}=\{{\left\{I_{k}^{\text{loc}}\right\}}_{k=0}^{t},{\left\{I_{k}^{\text{imp}}\right\}}_{k=0}^{t-1}\}$. In this expression, the parameters *α* > 0 and *β* > 0 are the shape and rate parameters of the gamma prior distribution for *R_t_*. The function gamma(*x*, *a*, *b*) corresponds to the probability density function of a gamma distribution with shape parameter *a* and rate parameter *b*, so that

$$\text{gamma}(x,a,b)=\frac{b^{a}}{\Gamma (a)}x^{a-1}\text{exp}(-bx).$$

The inferred posterior, *p*(*R_t_*|**w**, ε, ***I***_≤_***_t_***), is based on local infectees appearing in the incidence data in the estimation window [*t* − *τ*, *t*], infected by local or imported infectors appearing in the incidence data at any time in [0, *t* − 1]. Estimates of *R_t_* at successive time steps are generated by shifting the estimation window by one time step and repeating the inference procedure. The purpose of this estimation window (rather than estimating *R_t_* based on infectees appearing in the incidence time-series on day *t* alone) is to increase the smoothness of successive *R_t_* estimates, instead of inferring variations in *R_t_* due to the inherent randomness in the epidemiological system (or any other factor affecting the numbers of cases observed each day; for example, daily fluctuations in the proportion of cases that are reported). This comes at the cost of missing changes in transmission occurring at a fine temporal resolution [8].

### (b) Accounting for uncertainty in the serial interval distribution

The approach described above involves estimating *R_t_* using disease incidence time-series and an estimate of the serial interval distribution, accounting for differences in both the source location of infection and onwards transmission risk between local and imported cases. However, there is often significant uncertainty in the serial interval distribution. To account for this, we consider a scenario in which there is a set of equally plausible serial interval distributions, ${\left\{{\text{w}}^{\left(i\right)}\right\}}_{i=1}^{n}$. For a single value of *i*, the entries ${w_{s}}^{\left(i\right)}$ of the vector **w**^(***i***)^ correspond to the probability that the serial interval takes the value *s* days, conditional on **w**^(***i***)^ being the true serial interval distribution.

In our analyses of COVID-19 data, we use a set of equally plausible serial intervals, ${\left\{{\text{w}}^{\left(i\right)}\right\}}_{i=1}^{n}$, obtained from a previous study (see below). To account for this uncertainty in the serial interval distribution when estimating *R_t_*, we first estimate *R_t_* separately for each plausible serial interval distribution, **w**^(***i***)^, giving the conditional posterior distribution *p*(*R_t_*|**w**^(***i***)^, ε, ***I***_≤_***_t_***). We then combine these estimates to give a posterior distribution for *R_t_* accounting for this uncertainty by calculating

$$p(R_{t}\text{|}\varepsilon ,I_{\leq t})=\frac{1}{n}\overset{n}{\underset{i=1}{\sum }}p(R_{t}|{\text{w}}^{\left(i\right)},\varepsilon ,I_{\leq t}).$$

### (c) Data and parameterization

**Figure 2.**
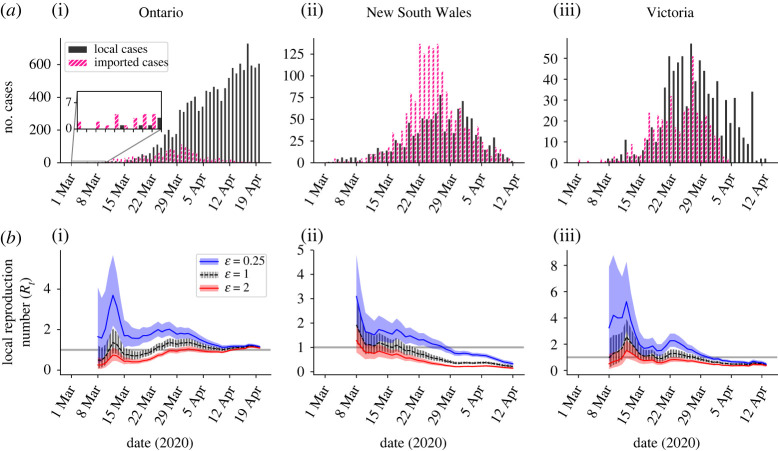
Inference of the local reproduction number (*R_t_*) under different assumptions about the relative transmission risk from imported and local cases. (*a*) The COVID-19 incidence time-series datasets used in our main analyses, for Ontario (i), New South Wales (ii) and Victoria (iii). Black bars represent the daily numbers of local cases, and pink bars represent the daily numbers of imported cases. (*b*) Inferred *R_t_* values for different assumed values of the relative transmission risk from an imported case compared with a local case (ε). The grey horizontal line represents the threshold *R_t_* = 1, and shaded regions represent the 95% central credible interval of the *R_t_* estimates. (Online version in colour.)

In our main analyses, we consider five disease incidence time-series datasets collected in different locations during the COVID-19 pandemic. The key feature of these datasets is that information is available which allows locally originating cases to be differentiated from those infected elsewhere. The datasets are as follows:

(i) Ontario, Canada (figure 2*a*(i)). Incidence data were obtained for the time period from 1 March to 20 April 2020 [33]. Cases were classified as imported if they reported travelling outside Ontario within 14 days prior to symptom onset. Cases with unknown recent travel status were assumed to have been infected locally.
(ii) New South Wales, Australia (figure 2*a*(ii)). Incidence data were obtained for the time period from 1 March to 13 April 2020. Cases were classified as imported if they were reported as ‘overseas acquired’ in the Australian national COVID-19 database (see [32] for further details). Cases with unknown origin were assumed to have been infected locally.
(iii) Victoria, Australia (figure 2*a*(iii)). Details as above for New South Wales.
(iv) Hong Kong (figure 4*a*(i)). Incidence data were obtained for the time period from 23 January to 24 March 2020 [34]. Cases were classified as imported if they were listed as ‘imported case, confirmed’ in the Hong Kong Department of Health COVID-19 database (see [35] for further details). All other cases were classified as local cases.
(v) Hainan Province, China (figure 4*a*(ii)). Incidence data were obtained for the time period from 22 January to 20 February 2020 [36]. Cases were classified as imported if they either reported travel outside Hainan Province in the 14 days prior to symptom onset or reported any recent travel to a known COVID-19 outbreak area. All other cases were classified as local cases.

We chose to analyse the first three datasets in the main text due to their differing outbreak trajectories in the time periods considered. Specifically, the Ontario dataset represents a growing outbreak, the New South Wales dataset represents a full outbreak wave with a large number of imported cases compared with local cases and the Victoria dataset represents a full outbreak wave with more local cases than imported ones. We chose to analyse the fourth and fifth datasets because further information was available from those locations with which it was possible to approximate the value of ε. This allowed us to demonstrate inference of *R_t_* in scenarios in which the relative transmission risk from imported and local cases is known.

In addition to our analyses in the main text, we considered datasets from other locations and display similar analyses in the electronic supplementary material, figures S1–S6; specifically, we considered COVID-19 disease incidence time-series datasets from five other Australian states, New Zealand and Hawaii, and we considered a disease incidence time-series dataset for MERS in Saudi Arabia in 2014–2015. The key feature of all these datasets is that information was available with which to classify cases as either local or imported. In the analysis of MERS in Saudi Arabia, imported cases were not those who had arrived from a geographically distinct location. Instead, in that analysis, imported cases were those who were likely to have been infected directly from the animal reservoir.

For the serial interval in all our analyses of COVID-19 incidence datasets, we considered an estimate for SARS-CoV-2 obtained by Nishiura *et al.* [37]. Specifically, those authors fitted a log-normal distribution to data from known infector–infectee transmission pairs using Markov chain Monte Carlo (MCMC), thereby obtaining a set of equally plausible possible serial interval distributions. We considered the set of serial interval distributions obtained by Nishiura *et al.* [37] using both certain and probable infector–infectee pairs while accounting for right-truncation (i.e. the possibility that a dataset detailing infector–infectee pairs observed when the outbreak is ongoing excludes some transmissions with longer serial intervals that have not yet occurred). For our inference procedure, we used *n* = 1000 randomly selected MCMC iterations from their analysis, where each iteration characterizes a continuous distribution. Since our approach considers the number of new cases each day, we require a discrete serial interval distribution. We therefore ‘discretized’ the continuous distributions into daily time steps using the method described by Cori *et al.* [8] (see web appendix 11 of that article). The set of *n* = 1000 serial interval distributions used in our analysis (i.e. ${\left\{{\text{w}}^{\left(i\right)}\right\}}_{i=1}^{n}$) is shown in the electronic supplementary material, figure S7.

We fixed the parameters of the gamma distributed prior for *R_t_* so that both the mean and standard deviation were equal to five (to do this, we chose *α* = 1 and *β* = 0.2). The rationale for this choice is that a large standard deviation ensures that the prior is relatively uninformative, while a high mean ensures that the outbreak is unlikely to be determined as under control (*R_t_* < 1) unless there is substantial evidence from the data supporting this conclusion. In all of our analyses of COVID-19 incidence data, *R_t_* was estimated using a weekly sliding window, so that *τ* = 6 days. In the figures, the posterior distribution for *R_t_* shown on day *t* is based on a sliding window that ends on day *t* (i.e. the sliding window [*t* − *τ*, *t*]).

### (d) Correctness and reproducibility of results

We followed a range of software development practices to guard against coding errors and to ensure code reusability: these included collaborative coding using Github to manage merging of code via pull requests, unit testing of functions and classes (with 100% test coverage) and continuous integration testing. To ensure reproducibility of results, all analyses for this paper can be rerun by cloning our Github repository (https://github.com/SABS-R3-Epidemiology/transmission-heterogeneity-results) and executed via a single command from the terminal.

## 3. Results

### (a) Effect of the relative transmission risk on estimates of *R_t_*

To explore how different assumptions about the relative transmission risk from imported and local cases affect *R_t_* estimates, we initially applied our method to data from the first three locations described in Methods (figure 2). We considered three different assumptions about the relative transmission risk. First, we assumed that imported cases were each expected to generate fewer infections than local cases (ε = 0.25; figure 2*b*—blue). Second, we assumed instead that imported cases were each expected to generate more infections than local cases (ε = 2; figure 2*b*—red). Third, we made the standard assumption [9] that the transmission risk from each local case was identical to the transmission risk from each imported case (ε = 1; figure 2*b*—black). These analyses highlight that different assumed values of ε lead to different inferred *R_t_* values. As might be expected, assuming larger values of ε leads to smaller estimated values of *R_t_*, since more transmission is then attributed to imported cases rather than local cases.

**Figure 3.**
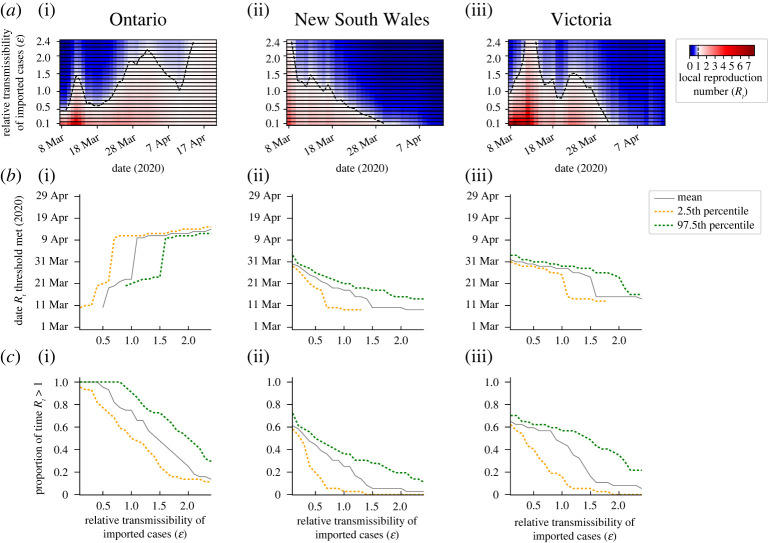
Implications of differences in the assumed relative transmission risk from imported and local cases on policymaking. (*a*) Inferred mean *R_t_* values for different values of the relative transmissibility of imported cases compared with local cases (ε). (*b*) Dates on which the estimated values of *R_t_* cross policy-relevant thresholds (in scenarios where the thresholds are crossed at some stage in the outbreak; otherwise dates are not plotted). For Ontario (i), the date shown represents the first date when the estimated *R_t_* value is above one and remains above one for the remainder of the time period considered (until 20 April 2020). This represents the first date when the outbreak is not inferred to be under control for the remainder of the time period. For New South Wales (ii) and Victoria (iii), the date shown represents the first date on which the estimated *R_t_* value is below one and remains so for the remainder of the time period considered (until 13 April 2020). This represents the first date on which the outbreak could be concluded as being under control for the remainder of the time period. (*c*) The proportion of the time periods considered for which the inferred *R_t_* values are above one (so the outbreak is not inferred to be under control). In (*b*,*c*), results are shown for the mean values of the posterior for *R_t_* (grey), and well as for the 2.5th (yellow dotted) and 97.5th (green dotted) percentile values of the posterior for *R_t_* (which span the 95% central credible interval). (Online version in colour.)

We then went on to consider the implications for public health policy of differences in the relative transmissibility of imported and local cases. For the dataset from Ontario (figure 2*a*(i)), the numbers of local cases broadly increased throughout the time period considered. A key question in that setting is ‘Is *R_t_* > 1?’, since this determines whether sustained local transmission is likely to occur. If so, fast detection that *R_t_* > 1 is crucial to allow interventions to be introduced quickly to prevent further exponential growth of the outbreak. In figure 3*a*(i), posterior mean estimates of *R_t_* each day from 8 March to 20 April 2020 are shown for a range of values of ε. The first date on which the mean estimate of *R_t_* is above one and remains above one thereafter is shown for different values of ε in figure 3*b*(i) (grey). This indicates that a smaller assumed value of ε leads to an earlier conclusion that *R_t_* is greater than one for this dataset. The proportion of the period considered for which the mean *R_t_* estimate is above one also depends on the assumed value of ε (figure 3*c*(i)).

While a policymaker may choose to strengthen control measures when the mean estimate of *R_t_* increases above one, a more risk-averse choice could be to conclude that the outbreak is not under control if an upper percentile of the posterior distribution of *R_t_* exceeds one. For example, for the Ontario dataset, when ε = 1.2, the mean estimate of *R_t_* is (and remains) above one from 11 April 2020 onwards (figure 3*b*(i), grey), whereas the 97.5th percentile estimate of *R_t_* remains above one from the earlier date of 23 March 2020 onwards (figure 3*b*(i), green dashed). By using an approach like the one described here, policymakers can adjust their decision making according to their chosen level of risk aversion. This simply involves specifying the percentile value of *R_t_* to track to guide decision making regarding strengthening and relaxing public health measures.

During the COVID-19 pandemic, public health measures have been relaxed in many regions and countries when the outbreak has been assessed as being under control. We therefore considered the incidence dataset from New South Wales and estimated when policymakers could conclude that *R_t_* had fallen below one (figure 3*b*(ii)). In this scenario, a larger assumed value of ε led to an earlier date on which *R_t_* was assessed to be below one (and remained below one thereafter). In order for policymakers to be more certain that *R_t_* is below one when relaxing restrictions, one possibility is to conclude that *R_t_* is below one when a high percentile value of the posterior for *R_t_* has fallen below one. For example, for this dataset, if the mean estimate of *R_t_* is considered and the value ε = 1.2 is assumed, then *R_t_* is inferred to fall and remain below one on 15 March 2020 (figure 3*b*(ii), grey), whereas if instead the 97.5th percentile estimate of *R_t_* is considered, then *R_t_* is inferred to fall below one on the later date of 19 March 2020 (figure 3*b*(ii), green dashed).

As the final component of these analyses, we considered the disease incidence time-series dataset from Victoria and repeated the analysis that we conducted for the dataset from New South Wales. We found that, if a high value of ε is assumed, then the outbreak is inferred to be under control (*R_t_* < 1) for the majority of the time period under consideration (figure 3*c*(iii)). However, if instead the value of ε is lower, then *R_t_* may be estimated to be greater than one early in the outbreak. For small values of ε, so that initial estimated values of *R_t_* are high, the most policy-relevant question may again be to determine when *R_t_* has fallen below one (figure 3*b*(iii)).

### (b) Realistic values of the relative transmission risk

In the analyses presented in §3a, we demonstrated clearly that the assumed relative transmission risk between imported and local cases affects *R_t_* estimates, impacting policy-relevant conclusions drawn from disease incidence time-series data. The relative transmission risk may differ between settings. In some scenarios, it may be possible to inform estimates of ε with real-world data. Here we provide two examples, in the context of SARS-CoV-2 transmission in Hong Kong and Hainan Province (the fourth and fifth disease incidence time-series datasets described in Methods). Additional possible approaches for estimating the value of ε are described in the Discussion.

**Figure 4.**
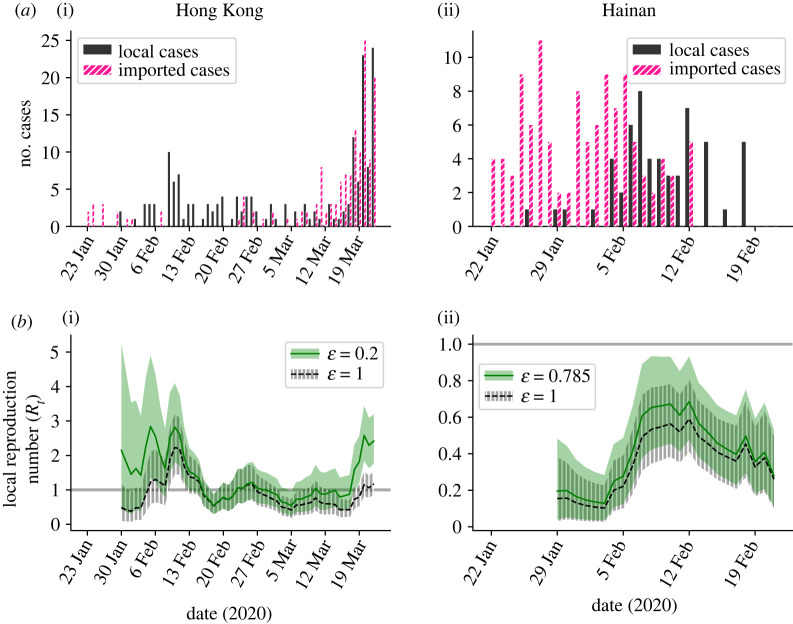
Inference of the local reproduction number (*R_t_*) for estimated values of the relative transmission risk from imported and local cases. (*a*) The COVID-19 incidence time-series datasets used in our main analyses, for Hong Kong (i) and Hainan Province, China (ii). Black bars represent the daily numbers of local cases, and pink bars represent the daily numbers of imported cases. (*b*) Inferred *R_t_* values for different assumed values of the relative transmission risk from an imported case compared with a local case (ε), for Hong Kong (i) and Hainan Province (ii). The grey horizontal line represents the threshold *R_t_* = 1, and shaded regions represent the 95% central credible interval of the *R_t_* estimates. The values ε = 0.2 for Hong Kong and ε = 0.785 for Hainan were estimated from alternative data sources, as described in the text. (Online version in colour.)

First, we considered the dataset from Hong Kong (figure 4*a*(i)). A previous study [35] reconstructed the transmission network of cases in that region (between 23 January 2020 and 8 January 2021; although in principle a similar analysis could be conducted at a smaller spatial scale for shorter time periods, as would likely be most useful for early real-time estimation of *R_t_*), inferring the ‘outdegree’ of imported and local cases. Based on the aggregated data shown in table 1 of that study, the mean outdegree was 0.74 for imported cases and 3.68 for local cases, which corresponds to a value of ε = 0.2. We therefore compared estimated values of *R_t_* for ε = 0.2 (figure 4*b*(i), green) with analogous estimates under the standard assumption that ε = 1 (figure 4*b*(i), black). Since a value of ε = 0.2 leads to less transmission being attributed to imported infections than when ε = 1, estimated values of *R_t_* are higher when ε = 0.2. In terms of decision making during an ongoing outbreak, time periods when the mean estimated value of *R_t_* is greater than one for ε = 0.2 and less than one for ε = 1 may be particularly concerning. In these periods, the outbreak might erroneously be inferred as being under control if the incorrect assumption that ε = 1 is made. In the analysis shown in figure 4*b*(i), this is the case for 20.8% of the time period considered. Of course, similarly to the analyses presented in §3a, analogous analyses could be performed based on different percentile estimates of *R_t_* rather than the mean estimated value.

Second, we considered the dataset from Hainan Province, China. A previous study [36] compared the epidemiological features of imported and local cases in that province and found that imported cases tended to belong to older age groups than local cases. We applied a contact matrix for China [38] to the age distributions of imported and local cases, and thus estimated the expected number of contacts per day for imported cases (10.5) and local cases (13.4). To approximate the value of ε, we divided the expected number of contacts per day for imported cases by the analogous value for local cases, giving ε = 0.785. We then compared estimates of *R_t_* for that more realistic value of ε = 0.785 (figure 4*b*(ii), green) with estimates of *R_t_* under the standard assumption that ε = 1 (figure 4*b*(ii), black). Since a value of ε = 1 is only slightly larger than ε = 0.785, and the data from Hainan Province suggest only limited local transmission, we found that incorrectly assuming that ε = 1 did not have a substantial effect on inferred *R_t_* values for this dataset.

## 4. Discussion

Summary statistics for tracking pathogen transmissibility are increasingly used during infectious disease outbreaks to guide decision making. Throughout the COVID-19 pandemic, *R_t_* has been estimated in regions and countries worldwide (see e.g. [7]). This metric is useful and straightforward to interpret, corresponding to the number of individuals that one infected host is expected (on average) to go on to infect. As well as providing information about whether an outbreak is growing or declining, the value of *R_t_* can be used to determine the proportion of transmissions that must be prevented for a growing outbreak to decline.

In this article, we have presented a modified version of the commonly used Cori method for inferring *R_t_* [8,9]. We have accounted for different transmission risks from local and imported cases, rather than assuming that the transmission risk is identical for individuals in these groups. We provide an accompanying online software tool for estimating *R_t_* (https://sabs-r3-epidemiology.github.io/branchpro) where users can upload their own data (disease incidence time-series and an estimate of the serial interval distribution—or multiple equally plausible serial interval distributions as described in Methods). We have conducted a systematic analysis of the dependence of inferred *R_t_* values on the assumed relative transmission risk from an imported case compared with a local case (ε; see figures 2 and 3). We also considered examples in which it was possible to approximate the value of ε from other data sources (figure 4). In general, larger assumed values of ε lead to smaller *R_t_* estimates. This is important, since assuming an unrealistically high value of ε may lead to the outbreak being falsely determined as under control. When an outbreak is ongoing, we have shown that the speed at which local transmission can be inferred as being either under control or not depends on the assumed value of ε. This dependence on ε demonstrates clearly that whether or not an outbreak is under control cannot always be inferred accurately from summary statistics that do not account for differences in the transmission risk between imported and local cases (e.g. the growth rate of overall cases). We have also shown how different percentile estimates of *R_t_* can be used to guide decision making, according to the policymaker's level of acceptable risk.

A previous approach for estimating *R_t_* allows infectees to have been infected either within or outside the local population [9]. However, in that framework, an assumption is made that the transmission risk from a local case is identical to the analogous risk from an imported case. The potential for different transmission risks from imported and local cases has implications for optimizing interventions, since if the risk of transmission is predominantly from imported cases, then travel restrictions and interventions that prevent transmissions from imported cases (e.g. quarantine of incoming travellers) may be the optimal measures. If instead the transmission risk is highest from local cases, then interventions such as social distancing and face coverings that reduce transmission from all infected individuals in the population may be necessary. In scenarios in which a novel pathogen variant is being imported into a new location from somewhere it is already widespread, the composition of variants causing local and imported cases might affect the relative transmission risk [39]. However, we note that in our modelling framework it is only the imported cases themselves that are assumed to represent a different transmission risk (rather than all infected individuals in a chain of transmission starting with an imported case).

A recent, closely related study by Tsang *et al.* [31] involved estimating independent *R_t_* values for local and imported cases throughout an outbreak. A benefit of that approach is that it does not require an assumption to be made about the relative transmission risk from each imported case than from each local case. However, there are substantial logistical challenges to estimating independent *R_t_* values for local and imported cases: this requires local cases who were infected by other local cases to be distinguished from those who were infected by imported cases. This may be possible either on a small scale or in locations with extensive contact tracing [31,40], but, in many situations, it is infeasible. In the absence of data with which to estimate *R_t_* for local and imported cases independently, and without known changes in the relative transmission risk from imported compared with local cases, then assuming a constant relative transmission risk between the two types of case as we have done seems reasonable. To obtain an idea about whether the relative transmission risk (i.e. the parameter ε in our model) is likely to be less than or greater than one, we considered examples in which we approximated ε using either a reconstructed transmission network or the age characteristics of local and imported cases (figure 4). In both examples that we considered, the estimated value of ε was less than one, suggesting a lower transmission risk from each imported case than from each local case in those settings. Other approaches for inferring ε are also possible. One way to estimate ε is to analyse data containing both local and imported cases in small-scale settings in which infector–infectee transmission pairs can be identified or estimated, such as household or contact tracing studies. Another option might be to perform forwards contact tracing on imported cases at a single stage of the outbreak. If the value of ε*R_t_* can be estimated from the contact tracing data at that stage, ε could then be estimated from the population-level incidence data. The contribution of imported cases to transmission is likely to vary by the time in the outbreak and by location [32,41]. In principle, estimates of ε could be updated based on the latest available contact tracing data.

In our main analyses, we have considered scenarios in which imported cases are individuals who have been infected in other geographical locations. However, an imported case may be defined as any case with an infection source outside the local host population. In the electronic supplementary material, we consider an analysis of MERS cases in Saudi Arabia in 2014–2015 (electronic supplementary material, figures S5 and S6), where cases are likely to have arisen both via human-to-human transmission and from an animal reservoir (specifically, from dromedary camels [42]). In that analysis, imported cases are assumed to be those reporting regular contacts with camels. It is possible that those individuals typically live in less densely populated areas than individuals who do not have regular contacts with camels, meaning that the relative risk of an imported case transmitting the virus is lower on average than the analogous risk from a local case. Like our analyses of COVID-19 datasets, our analysis of the MERS incidence data illustrates that assumptions about the relative transmission risk between local and imported cases can affect estimates of *R_t_* and conclusions about whether or not local human-to-human transmission is under control.

We also conducted an additional supplementary analysis in which we generated synthetic epidemic datasets and investigated further the conditions under which mischaracterizing the relative transmissibility of imported and local cases affects estimates of *R_t_* substantially. Specifically, we generated synthetic data for different values of ε and different strengths of local transmission. We calculated the error in estimates of *R_t_* if the standard assumption that ε = 1 is made (electronic supplementary material, figure S8). This suggests that the largest errors occur when the relative transmissibility of imported (compared with local) cases differs substantially from one, and when imported cases represent a high proportion of the overall cases observed in the population.

In the research that we have presented, we sought to explore the relationship between heterogeneities in the onwards transmission risk between different groups of infectious individuals and inferred values of *R_t_*. Practical applications of this approach should consider incorporating additional features into the modelling framework. An important consideration when assessing pathogen transmissibility during outbreaks is that *R_t_* represents the average number of onwards infections over multiple infected individuals and transmission events. However, different infected individuals may generate very different numbers of infections [10,43–45]. The potential for super-spreading events at which large numbers of infections occur could be built into the underlying transmission model and into the resulting *R_t_* estimates, although it may then be impossible to generate an analytic expression for the posterior for *R_t_*. We sought to demonstrate the general principle that population heterogeneity can affect estimates of *R_t_*. To do this as simply as possible, we used a model with only two groups of infected hosts (i.e. local and imported cases) and assumed that individuals are classified accurately as either local or imported. However, the classification of hosts into distinct groups may be imperfect (as considered elsewhere in this theme issue [46]), and many different sources of heterogeneity exist within host populations. There may be substantial differences in the transmission risk between other subgroups of the population: for example, risk may vary by age [10,25,26] and vaccination status [29]. Geographically distinct populations could be linked in a transmission model, so that spatial heterogeneity in *R_t_* can be explored. In principle, compartmental models can be developed in which a range of different sources of heterogeneity are included, and *R_t_* may be estimated using those compartmental models. It might also be possible to include further sources of heterogeneity in a renewal equation framework as studied here. These possibilities represent interesting avenues for future research.

Here, we assumed that the data represent disease incidence time-series, and that the serial interval (the time between successive symptomatic cases in a transmission chain) is always positive. In reality, pre-symptomatic infections occur, and serial intervals may take negative values [47–49] with infectors developing symptoms after some of the individuals who they infect. While the assumption of a positive valued serial interval distribution has been made in many previous studies in which *R_t_* has been estimated for different pathogens, this issue can be avoided by using the incidence of infections and the generation time distribution [47,49,50] rather than the incidence of cases and the serial interval distribution [11]. The subtle difference here is that incidence time-series of cases do not reflect the times at which individuals were first infected, but instead reflect the times at which individuals were recorded as infected (which occurs after infection, for example when individuals display symptoms). Use of the incidence of infections and the generation time distribution may require the incidence of infections to be inferred from the incidence of cases, for example using an assumed incubation period distribution and the Richardson–Lucy deconvolution technique [51]. We note that the serial interval distribution may be different to the generation time distribution (specifically, pre-symptomatic transmission can lead to shorter serial intervals than generation times [49]). Another potential extension to our research is incorporation of different serial interval (or generation time) distributions for local and imported cases [40], particularly given that part of an imported case's infectious period may occur before they enter the local population. Reconstructed transmission networks might provide insights into these distributions.

More broadly, we note that *R_t_* is only one summary statistic for tracking changes in transmission during an infectious disease outbreak. This metric does not provide information about the speed of the outbreak, which is better measured by the growth rate of cases [52,53,54]. Furthermore, current incidence of reported cases, hospitalizations and deaths are also key inputs to policy decisions. For example, an outbreak with *R_t_* close to one is likely to have more detrimental impacts if case numbers are high compared with if case numbers are low. Nonetheless, *R_t_* has been useful for guiding interventions during the COVID-19 pandemic, in combination with these other statistics. We therefore contend that studies that improve understanding of the impacts of factors affecting *R_t_* estimates, such as heterogeneity in the onwards transmission risk between different infectious hosts, are valuable and an important component of preparedness for future outbreaks.

## Data Availability

The user-friendly web interface for estimating *R_t_* while accounting for different transmission risks from local and imported cases can be found at https://sabs-r3-epidemiology.github.io/branchpro. All data and computing scripts required to reproduce the results presented here are available at https://github.com/SABS-R3-Epidemiology/transmission-heterogeneity-results. The source code of the branchpro Python package, which we developed to perform the inference presented in this article, is available at https://github.com/SABS-R3-Epidemiology/branchpro. No restrictions exist on data availability. The data are provided in the electronic supplementary material [55].

## References

[1] Brooks-Pollock E, Danon L, Jombart T, Pellis L. 2021 Modelling that shaped the early COVID-19 pandemic response in the UK. Phil. Trans. R. Soc. B **376**, 20210001. (10.1098/rstb.2021.0001) 34053252 PMC8165593

[2] Thompson RN. 2020 Epidemiological models are important tools for guiding COVID-19 interventions. BMC Med. **18**, 152. (10.1186/s12916-020-01628-4) 32448247 PMC7246085

[3] Davies NG *et al.*, CMMID COVID-19 working group. 2020 The effect of non-pharmaceutical interventions on COVID-19 cases, deaths and demand for hospital services in the UK: a modelling study. Lancet Public Health **5**, e375-e385. (10.1016/S2468-2667(20)30133-X) 32502389 PMC7266572

[4] Moore S, Hill EM, Dyson L, Tildesley MJ, Keeling MJ. 2020 Modelling optimal vaccination strategy for SARS-CoV-2 in the UK. PLoS Comput. Biol. **17**, e1008849. (10.1371/journal.pcbi.1008849) PMC8101958 33956791

[5] Dehning J, Zierenberg J, Spitzner EP, Wibral M, Neto JP, Wilczek M, Priesemann V. 2020 Inferring change points in the spread of COVID-19 reveals the effectiveness of interventions. Science **369**, eabb9789. (10.1126/science.abb9789) 32414780 PMC7239331

[6] Birrell P, Blake J, Van Leeuwen E, Gent N, De Angelis D. 2020 Real-time nowcasting and forecasting of COVID-19 dynamics in England: the first wave. Phil. Trans. R. Soc. B **376**, 20200279. (10.1098/rstb.2020.0279) PMC8165585 34053254

[7] Abbott S *et al.* 2020 Estimating the time-varying reproduction number of SARS-CoV-2 using national and subnational case counts. Wellcome Open Res. **5**, 112. (10.12688/wellcomeopenres.16006.1)

[8] Cori A, Ferguson NM, Fraser C, Cauchemez S. 2013 A new framework and software to estimate time-varying reproduction numbers during epidemics. Am. J. Epidemiol. **178**, 1505-1512. (10.1093/aje/kwt133) 24043437 PMC3816335

[9] Thompson RN *et al.* 2019 Improved inference of time-varying reproduction numbers during infectious disease outbreaks. Epidemics **29**, 100356. (10.1016/j.epidem.2019.100356) 31624039 PMC7105007

[10] Thompson RN *et al.* 2020 Key questions for modelling COVID-19 exit strategies. Proc. R. Soc. B **287**, 20201405. (10.1098/rspb.2020.1405) PMC7575516 32781946

[11] Gostic KM *et al.* 2020 Practical considerations for measuring the effective reproductive number, *R_t_*. PLoS Comput. Biol. **16**, e1008409. (10.1371/journal.pcbi.1008409) 33301457 PMC7728287

[12] White LF, Moser CB, Thompson RN, Pagano M. 2020 Statistical estimation of the reproductive number from case notification data. Am. J. Epidemiol. **190**, 611-620. (10.1093/aje/kwaa211) PMC8244992 33034345

[13] Nishiura H, Chowell G. 2009 The effective reproduction number as a prelude to statistical estimation of time-dependent epidemic trends. In Mathematical and statistical estimation approaches in epidemiology, pp. 103-121. Dordrecht, The Netherlands: Springer.

[14] Cowling BJ, Lau MSY, Ho LM, Chuang SK, Tsang T, Liu SH, Leung PY, Lo SV, Lau EH. 2010 The effective reproduction number of pandemic influenza: prospective estimation. Epidemiology **21**, 842-846. (10.1097/EDE.0b013e3181f20977) 20805752 PMC3084966

[15] Cauchemez S, Boëlle PY, Donnelly CA, Ferguson NM, Thomas G, Leung GM, Hedley AJ, Anderson RM, Valleron AJ. 2006 Real-time estimates in early detection of SARS. Emerg. Infect. Dis. **12**, 110-113. (10.3201/eid1201.050593) 16494726 PMC3293464

[16] Cheng Q, Liu Z, Cheng G, Huang J. 2020 Heterogeneity and effectiveness analysis of COVID-19 prevention and control in major cities in China through time-varying reproduction number estimation. Sci. Rep. **10**, 21953. (10.1038/s41598-020-79063-x) 33319859 PMC7738538

[17] Ackland GJ, Ackland JA, Antonioletti M, Wallace DJ. 2022 Fitting the reproduction number from UK coronavirus case data and why it is close to 1. Phil. Trans. R. Soc. A **380**, 20210301. (10.1098/rstb.2021.0301) PMC9376721 35965470

[18] UK Government. 2021 The R value and growth rate. See https://www.gov.uk/guidance/the-r-value-and-growth-rate.

[19] Fraser C. 2007 Estimating individual and household reproduction numbers in an emerging epidemic. PLoS ONE **2**, e758. (10.1371/journal.pone.0000758) 17712406 PMC1950082

[20] Obadia T, Haneef R, Boëlle PY. 2012 The R0 package: a toolbox to estimate reproduction numbers for epidemic outbreaks. BMC Med. Inform. Decis. Mak. **12**, 147. (10.1186/1472-6947-12-147) 23249562 PMC3582628

[21] Vegvari C *et al.* 2021 Commentary on the use of the reproduction number R during the COVID-19 pandemic. Stat. Methods Med. Res. **1**, 09622802211037079. (10.1177/09622802211037079) PMC9277711 34569883

[22] Wallinga J, Teunis P. 2004 Different epidemic curves for severe acute respiratory syndrome reveal similar impacts of control measures. Am. J. Epidemiol. **160**, 509-516. (10.1093/aje/kwh255) 15353409 PMC7110200

[23] Ali ST, Wang L, Lau EHY, Xu XK, Du Z, Wu Y, Leung GM, Cowling BJ. 2020 Serial interval of SARS-CoV-2 was shortened over time by nonpharmaceutical interventions. Science **369**, 1106-1109. (10.1126/science.abc9004) 32694200 PMC7402628

[24] Ladhani SN *et al.* 2020 Increased risk of SARS-CoV-2 infection in staff working across different care homes: enhanced COVID-19 outbreak investigations in London care homes. J. Infect. **81**, 621-624. (10.1016/j.jinf.2020.07.027) 32735893 PMC7387283

[25] Davies NG, Klepac P, Liu Y, Prem K, Jit M, Eggo RM, CMMID COVID-19 Working Group. 2020 Age-dependent effects in the transmission and control of COVID-19 epidemics. Nat. Med. **26**, 1205-1211. (10.1038/s41591-020-0962-9) 32546824

[26] Keeling MJ *et al.* 2021 Predictions of COVID-19 dynamics in the UK: short-term forecasting and analysis of potential exit strategies. PLoS Comput. Biol. **17**, e1008619. (10.1371/journal.pcbi.1008619) 33481773 PMC7857604

[27] Lovell-Read FA, Shen S, Thompson RN. 2022 Estimating local outbreak risks and the effects of non-pharmaceutical interventions in age-structured populations: SARS-CoV-2 as a case study. J. Theor. Biol. **535**, 110983. (10.1016/j.jtbi.2021.110983) 34915042 PMC8670853

[28] Pooley CM, Doeschl-Wilson AB, Marion G. 2022 Estimation of age-stratified contact rates during the COVID-19 pandemic using a novel inference algorithm. Phil. Trans. R. Soc. A **380**, 20210298. (10.1098/rsta.2021.0298) PMC9376725 35965466

[29] Keeling MJ, Dyson L, Hill E, Moore S, Tildesley MJ. 2021. Road map scenarios and sensitivity: Step 4. [cited 4 Aug 2021]. See https://assets.publishing.service.gov.uk/government/uploads/system/uploads/attachment_data/file/993358/s1288_Warwick_RoadMap_Step_4.pdf.

[30] Sachak-Patwa R, Byrne HM, Dyson L, Thompson RN. 2021 The risk of SARS-CoV-2 outbreaks in low prevalence settings following the removal of travel restrictions. Comms. Med. **1**, 39. (10.1038/s43856-021-00038-8) PMC9053223 35602220

[31] Tsang TK, Wu P, Lau EHY, Cowling BJ. 2021 Accounting for imported cases in estimating the time-varying reproductive number of COVID-19 in Hong Kong. J. Infect. Dis. **224**, 783-787. (10.1093/infdis/jiab299) 34086944 PMC8244742

[32] Price DJ *et al.* 2020 Early analysis of the Australian COVID-19 epidemic. eLife **9**, e58785. (10.7554/eLife.58785) 32788039 PMC7449695

[33] Government of Ontario. 2021 COVID-19 data: likely source of infection. See https://covid-19.ontario.ca/data/likely-source-infection.

[34] Hong Kong Department of Health. 2021 Latest local situation of COVID-19. See https://data.gov.hk/en-data/dataset/hk-dh-chpsebcddr-novel-infectious-agent/resource/ec4b49af-83e0-4c71-a3ba-14120e453b9d.

[35] Liu Y, Gu Z, Liu J. 2021 Uncovering transmission patterns of COVID-19 outbreaks: a region-wide comprehensive retrospective study in Hong Kong. EClinicalMedicine **36**, 100929. (10.1016/j.eclinm.2021.100929) 34124628 PMC8179759

[36] Wu B *et al.* 2020 Compare the epidemiological and clinical features of imported and local COVID-19 cases in Hainan, China. Infect. Dis. Poverty **9**, 143. (10.1186/s40249-020-00755-7) 33076968 PMC7569564

[37] Nishiura H, Linton NM, Akhmetzhanov AR. 2020 Serial interval of novel coronavirus (COVID-19) infections. Int. J. Infect. Dis. **93**, 284-286. (10.1016/j.ijid.2020.02.060) 32145466 PMC7128842

[38] Prem K, Cook AR, Jit M. 2017 Projecting social contact matrices in 152 countries using contact surveys and demographic data. PLoS Comp. Biol. **13**, e1005697. (10.1371/journal.pcbi.1005697) PMC5609774 28898249

[39] Challen R *et al.* 2021 Early epidemiological signatures of novel SARS-CoV-2 variants: establishment of B.1.617.2 in England. *medRxiv*. (10.1101/2021.06.05.21258365)

[40] Li M, Liu K, Song Y, Wang M, Wu J. 2021 Serial interval and generation interval for imported and local infectors, respectively, estimated using reported contact-tracing data of COVID-19 in China. Front. Public Health **8**, 577431. (10.3389/fpubh.2020.577431) 33490015 PMC7821042

[41] Russell TW, Wu JT, Clifford S, Edmunds WJ, Kucharski AJ, Jit M, Centre for the Mathematical Modelling of Infectious Diseases COVID-19 working group. 2021 Effect of internationally imported cases on internal spread of COVID-19: a mathematical modelling study. Lancet Public Health **6**, e12-e20. (10.1016/S2468-2667(20)30263-2) 33301722 PMC7801817

[42] Haagmans BL *et al.* 2014 Middle East respiratory syndrome coronavirus in dromedary camels: an outbreak investigation. Lancet Infect. Dis. **14**, 140-145. (10.1016/S1473-3099(13)70690-X) 24355866 PMC7106553

[43] Endo A, Abbott S, Kucharski AJ, Funk S, CMMID COVID-19 working group. 2020 Estimating the overdispersion in COVID-19 transmission using outbreak sizes outside China. Wellcome Open Res. **5**, 67. (10.12688/wellcomeopenres.15842.3) 32685698 PMC7338915

[44] Lloyd-Smith JO, Schreiber SJ, Kopp PE, Getz WM. 2005 Superspreading and the effect of individual variation on disease emergence. Nature **438**, 355-359. (10.1038/nature04153) 16292310 PMC7094981

[45] Akhmetzhanov AR, Jung SM, Cheng HY, Thompson RN. 2021 A hospital-related outbreak of SARS-CoV-2 associated with variant Epsilon (B.1.429) in Taiwan: transmission potential and outbreak containment under intensified contact tracing, January–February 2021. Int. J. Infect. Dis. **110**, 15-20. (10.1016/j.ijid.2021.06.028) 34146689 PMC8214728

[46] Li W, Bulekova K, Gregor B, White LF, Kolaczyk ED. 2022 Estimation of local time-varying reproduction numbers in noisy surveillance data. Phil. Trans. R. Soc. A **380**, 20210303. (10.1098/rsta.2021.0303) PMC9376722 35965456

[47] Hart WS, Maini PK, Thompson RN. 2021 High infectiousness immediately before COVID-19 symptom onset highlights the importance of continued contact tracing. eLife **10**, e65534. (10.7554/eLife.65534) 33899740 PMC8195606

[48] Du Z, Xu X, Wu Y, Wang L, Cowling BJ, Meyers LA. 2020 Serial interval of COVID-19 among publicly reported confirmed cases. Emerg. Infect. Dis. **26**, 1341-1343. (10.3201/eid2606.200357) 32191173 PMC7258488

[49] Hart WS, Miller E, Andrews NJ, Waight P, Maini PK, Funk S, Thompson RN. 2022 Generation time of the alpha and delta SARS-CoV-2 variants: an epidemiological analysis. Lancet Infect. Dis. **22**, 603-610. (10.1016/S1473-3099(22)00001-9) 35176230 PMC8843191

[50] Hart WS, Abbott S, Endo A, Hellewell J, Miller E, Andrews N, Maini PK, Funk S, Thompson RN. 2022 Inference of the SARS-CoV-2 generation time using UK household data. eLife **11**, e70767. (10.7554/eLife.70767) 35138250 PMC8967386

[51] Goldstein E, Dushoff J, Ma J, Plotkin JB, Earn DJD, Lipsitch M. 2009 Reconstructing influenza incidence by deconvolution of daily mortality time series. Proc. Natl Acad. Sci. USA **106**, 21829. (10.1073/pnas.0902958106) PMC2796142 20080801

[52] Dushoff J, Park SW. 2021 Speed and strength of an epidemic intervention. Proc. R. Soc. B **288**, 20201556. (10.1098/rspb.2020.1556) PMC8059560 33757359

[53] Pellis L *et al.* 2021 Challenges in control of Covid-19: short doubling time and long delay to effect of interventions. Phil. Trans. R. Soc. B **376**, 20200264. (10.1098/rstb.2020.0264) 34053267 PMC8165602

[54] Parag KV, Thompson RN, Donnelly CA 2022 Are epidemic growth rates more informative than reproduction numbers? J. Roy. Stat Soc. **1**, 1–11. (10.1111/rssa.12867) PMC9347870 35942192

[55] Creswell R *et al.* 2022 Heterogeneity in the onwards transmission risk between local and imported cases affects practical estimates of the time-dependent reproduction number. Figshare. (10.6084/m9.figshare.c.6070469) PMC9376709 35965464

